# The relation of nasopharyngeal colonization by *Streptococcus pneumoniae* in comorbid adults with unfavorable outcomes in a low-middle income country

**DOI:** 10.1371/journal.pone.0318320

**Published:** 2025-02-12

**Authors:** Juan Olivella-Gomez, Julián Lozada, Cristian C. Serrano-Mayorga, Lina Méndez-Castillo, Alejandro Acosta-González, André Emilio Viñán Garcés, Ingrid G. Bustos, Elsa D. Ibáñez-Prada, Yuli V. Fuentes, Ana M. Crispin, Erica Y. Garcia-Garcia, Eveling Santana, Diego F. Josa, Jorge Pulido Saenz, Gina Paola Rodíguez-Castaño, Jorge Alberto Rodríguez Orjuela, Diego Jaimes, Hervé Tettelin, Carlos J. Orihuela, Luis Felipe Reyes

**Affiliations:** 1 School of Medicine, Universidad de La Sabana, Chía, Colombia; 2 Unisabana Center for Translational Science, Universidad de La Sabana, Chía, Colombia; 3 Clínica Universidad de La Sabana, Chía, Colombia; 4 Biosciences PhD, Engineering Faculty, Universidad de La Sabana, Chía, Colombia; 5 Grupo de Investigación en Bioprospección (G.I.B.P.), Faculty of Engineering, Universidad de La Sabana, Chía, Colombia; 6 Fundación Clínica Shaio, Department of Clinical Laboratory and Pathology, Molecular Biology - Microbiology Area Bogotá, Bogotá, Colombia; 7 Renal Care Services Baxter, Chía, Colombia; 8 Department of Microbiology and Immunology, Institute for Genome Sciences, University of Maryland School of Medicine, Baltimore, Maryland, United States of America; 9 Department of Microbiology, University of Alabama at Birmingham, Birmingham, Alabama, United States of America; 10 Pandemic Sciences Institute, University of Oxford, Oxford, United Kingdom; Norbert Wiener University, PERU

## Abstract

**Purpose:**

*Streptococcus pneumoniae (Spn*) is the primary bacterial cause of lower respiratory tract infections (LRTI) globally, particularly impacting older adults and children. While *Spn* colonization in children is linked to LRTI, its prevalence, and consequences in adults with comorbidities remain uncertain. This study aims to provide novel data in that regard.

**Methods:**

This prospective study of outpatient adults with chronic diseases was conducted in Colombia. Data on demographics, vaccination, and clinical history was collected in a RedCap database. Nasopharyngeal aspirate samples were examined for *Spn* colonization using traditional cultures and quantitative—real time polymerase chain reaction (q-rtPCR). Patients were followed for 18 months, with colonization prevalence calculated and factors influencing colonization and its impact on clinical outcomes analyzed through logistic regressions.

**Results:**

810 patients were enrolled, with 10.1% (82/810) identified as colonized. The mean (SD) age was 62 years (±15), and 48.6% (394/810) were female. Major comorbidities included hypertension (52.2% [423/810]), cardiac conditions (31.1% [252/810]), and chronic kidney disease (17.4% [141/810]). Among all, 31.6% (256/810) received the influenza vaccine in the previous year, and 10.7% (87/810) received anti-*Spn* vaccines. Chronic kidney disease (OR 95% CI; 2.48 [1.01–6.15], p = 0.04) and chronic cardiac diseases (OR 95% CI; 1.62 [0.99–2.66], p = 0.05) were independently associated with *Spn* colonization. However, colonization was not associated with the development of LRTI (OR 95%CI; 0.64 [0.14–2.79], p = 0.55) or unfavorable outcomes (OR 95% CI;1.17 [0.14–2.79], p = 0.54) during follow-up.

**Conclusions:**

Chronic kidney and cardiac diseases are independently associated with *Spn* colonization. However, *Spn* colonization was not associated with LRTI/unfavorable outcomes in adult patients with chronic comorbidities in our cohort.

## Introduction

Lower respiratory tract infections (LRTI) are the leading infectious causes of mortality worldwide [1]. According to the Global Burden of Disease (GBD), LRTI etiology is most frequently attributed to microorganisms such as *Streptococcus pneumoniae (Spn)*, *Haemophilus influenzae* type b (Hib), influenza viruses, and respiratory syncytial virus (RSV), among others [2]. *S*. *pneumoniae* is the leading cause of morbidity and mortality due to respiratory infectious diseases worldwide [3]. Notably, the highest burden of pneumococcal disease (PD) has been documented in vulnerable populations, such as the extremes of age and those with chronic comorbid conditions [4]. Several risk factors have been linked with PD in adults, including advanced age, chronic medical comorbidities (i.e., heart disease, chronic lung disease, diabetes mellitus, cancer, and chronic renal disease), and immunocompromising conditions [5, 6]. However, it has been postulated in children that nasopharyngeal pneumococcal colonization (NPC) plays an important role [7].

Nasopharyngeal carriage is considered the first step towards invasive pneumococcal disease (IPD) in children [7]. This colonization has been proposed to be attributed to several mechanisms, including the evasion of mucus entrapment by capsular polysaccharide, biofilm formation, expression of adhesion proteins, inhibition of complement proteins, and release of bacteriocins to mediate competition with local microbiota [8]. It has been controversial to consider *Spn* NPC as a prerequisite for developing IPD [9]. However, some authors have found a link between the acquisition/carriage by *Spn* and the development of a respiratory diseases [10–14]. In adults, several risk factors for colonization have been described, such as smoking, living with children, and residence in a nursing home [15] yet the association between *Spn* NPC and IPD development in adults remains unclear and unexplored.

This study aims to determine the prevalence of *Spn* NPC and to explore if there is a relationship between *Spn* NPC and the development of unfavorable outcomes (i.e., development of community-acquired pneumonia (CAP), hospital admission or mortality due to infectious diseases) in adult patients with comorbidities. In addition, it examines factors associated with *Spn* NPC colonization and describes anti-*Spn* vaccination rates in patients with comorbidities based on current local guidelines.

## Materials and methods

This prospective multicenter cohort study was conducted in adult patients with chronic diseases from four outpatient clinics in Bogotá, Colombia. This retrospective cohort study was done following the strengthening of the reporting of observational studies in epidemiology (STROBE) guidelines [16] and the tenets of the Helsinki declaration. The study protocol and written informed consent were developed by the Translational Science in Infectious Diseases and Critical Care Medicine (TSID-CCM) research group from the Universidad de La Sabana. These documents were reviewed and authorized by the Institutional Review Board/Independent Ethics Committee (IRB/EC) of each participating institutions (CUS 01-20Feb2020, Shaio 301-26Aug2020, IPS-Clínicos 01-20Feb2020, and Baxter 05-01Sep2021). Ethical supervision ensured written informed consent was obtained from all study participants or their authorized representatives.

Inclusion criteria consisted of individuals aged 18 years or older with at least one chronic disease. Comorbidities are defined in S1 Table. Participants were actively attending one of the four participating centers, with recruitment beginning on December 2^nd^ 2020 at two centers, January 2^nd^ 2021 at the third, and September 13^th^ 2021 at the fourth. Recruitment was completed in March 17^th^ 2022, once the target sample size was reached. The exclusion criteria included patients with evidence of respiratory symptoms (e.g., rhinorrhea, fever, or expectoration) or diagnosis of community-acquired pneumonia (CAP) during the prior to 90 days according to the criteria of the American Thoracic Association (ATS)—Infectious Diseases Society of America (IDSA) [17]. Also, subjects admitted to the hospital during the previous 7 days of enrollment or limited to providing biological-type samples were excluded from the study.

### Definitions

A patient was considered *Spn* NPC positive if at least one of the two tests described below yielded a positive result. *Spn* NPC was defined as the identification of *Spn* either by conventional culture, confirmed through matrix-assisted laser desorption/ionization time-of-flight mass spectrometry (MALDI-TOF), or by the amplification with sigmoidal curve amplification of both the pneumolysin (*ply*) and autolysin (*lytA*) genes using quantitative real-time polymerase chain reaction (q-rtPCR) in nasopharyngeal aspirates (NPA), and evaluated based on the patient’s specific conditions and local guidelines for anti-*Spn* vaccination [18].

Finally, the unfavorable outcome variable was composed of patients who developed CAP by any cause, including viral pneumonia (CAP diagnosis was determined using the definition of IDSA/ATS guidelines during the follow-up [17] hospitalization due to an infectious cause, whether by *Spn* or other microorganisms or succumbed to pneumonia or another infectious cause at any point during the follow-up period (i.e., 6, 12 and 18 months after enrollment).

### Data collection and laboratory procedures

Data collection was carried out by a dedicated research assistant, who gathered sociodemographic details of medical history, including comorbidities, living conditions, and lifestyle habits. Participants were also required to provide a verifiable vaccination record at baseline. The data collection process used an Electronic Case Report Form (eCRF) developed within the Research Electronic Data Capture (REDCap) platform (version 8.11.11 provided by Vanderbilt University, Nashville, Tenn.), hosted by Universidad de Sabana. Follow-up data was collected by telephone interviews 6, 12, and 18 months after enrollment. This data included information on hospital admissions, reason for hospitalization, symptoms associated with hospitalization, and hospital center where care was received.

For all patients enrolled in the study after signing informed consent, trained nursing staff collected NPA samples with an 8 mm nelaton probe (Medex, INVIMA 2008DM—0001689 R2, Colombia) and 8–10 cc rinsing infusion of physiological saline solution (PSS) of sodium chloride 0. 9% (Baxter, Viaflex) at 10–15 cm in the posterior nostril of each participant according to the guidelines of the WHO (World Health Organization) Pneumococcal Carriage Working Group in 2013 [19].

In the laboratory, 100 *μ*L of nasopharyngeal aspirate (NPA) was immediately inoculated on blood agar and incubated at 37°C +/− 2°C for 24–48 hours in 5% CO2. Alpha-hemolytic colonies were selected, underwent optochin sensitivity testing, and sensitive strains were identified using (MALDI-TOF) mass spectrometry. “Suspected cases” were defined as colonization with *Spn* when alpha-hemolytic colonies showed optochin sensitivity > 14 mm [20]. “Confirmed cases” had MALDI-TOF scores > 1.8 [21]. Additionally, a q-rtPCR approach targeting *ply* and *lytA* genes was employed, adhering to specific criteria for pneumococcal colonization identification, including Ct < 35 [22–24] and characteristic melt curve profiles (S1 File) [25]. Information regarding conventional culture, primer selection, run cycle, and standard curve identification for *ply* and *lytA* gene identification and specific criteria for colonization identification can be found in the online supplement (S1 File).

Throughout the follow-up period, phone interviews were systematically conducted with each patient at 6, 12, and 18 months from the enrollment date unless their demise or dissent of participation was previously known. These interviews aimed to gather information on instances of hospitalization, including the cause and duration, and to inquire about the development of pneumonia. If pneumonia occurred, patients were asked to provide their medical records. Then, a detailed chart review assessed the identified etiology and details of antibiotic use, including the duration of therapy and clinical outcomes. In cases where the patient had passed away, details such as the date and cause of death were meticulously recorded. Vaccination status was updated if the patient had been vaccinated during the study period. The inquiry spanned the last 6 months preceding the call.

### Statistical analysis

Data were accessed for research purposes on the 13th of November 2023. A descriptive analysis was conducted using measures of central tendency (mean or median) and dispersion (standard deviation or interquartile range), depending on the data distribution, which was assessed using the Shapiro-Wilk test for quantitative variables. For qualitative variables, frequencies and percentages were calculated. The period prevalence of *Spn* NPC was determined by dividing the number of positive *Spn* NPC cases by the total number of subjects recruited during the study period. Demographic variables and comorbidities were compared between the positive *Spn* NPC and negative *Spn* NPC groups to assess differences, using the chi-square or Fisher’s exact test for categorical variables and the Student’s T-test or Mann-Whitney U test for continuous variables based on their distribution. Missing data was assessed through multiple imputation when variables relevant to the study had 5–20% of missing data, if the percentage was under 5 imputation was not performed; if missing data accounted for more than 20% variables were excluded.

A univariate analysis was performed to identify variables potentially related to *Spn* NPC and unfavorable outcomes. After univariate analysis a binary multivariate regression model was applied to identify factors associated with *Spn* NPC, with adjustments made for age to include demographic details upon admission. The stepwise logistic regression model included variables with a significance level of 0.20 or lower from the univariate analysis and with biological plausibility as well as absence of collinearity as identified in a directed acyclic diagram. Finally, a multivariate regression analysis over the 18-month follow-up period was performed to explore the impact of colonization on unfavorable as defined above. This model was adjusted for age, vaccination status, and renal replacement therapy to address potential selection bias due to the significant number of patients undergoing renal replacement therapy. Odds ratios (OR) and 95% confidence intervals (95% CI) were calculated from the final model’s coefficients. All statistical analyses were conducted using IBM SPSS Statistics for Mac, version 22.0 (Armonk, NY: I.B.M. Corp).

## Results

Between December 2020 and March 2022, 810 patients were enrolled, with 10.1% (82/810) identified as colonized through conventional culture and/or the q-rtPCR method. In the overall cohort, the average age of the study population was 62 years, with a standard deviation of ± 15 years, and females represented 48.6% (394/810) of the cohort. The predominant comorbidity among the studied population was hypertension 52% (423/810), followed by cardiac conditions 31% (252/810), chronic kidney disease (CKD)17.4% (141/810), and immunologic compromise 17.1% (139/810); all the other comorbid conditions are presented in Table 1. Regarding environmental factors, 13.1% (106/810) of the patients lived with small children, and 14.4% (117/810) were smokers. Other detailed demographic and clinical characteristics for the overall cohort and stratified by *Spn* colonization are provided in (S2 Table).

**Table 1:** Distribution of comorbidities in *Streptococcus pneumoniae* (*Spn*) colonized and no *Spn* colonized patients.

	Total patients sampled n = 810	Conventional Culture or q-rtPCR negative for *Spn* colonization n = (728, 89.9%)	Conventional Culture or q-rtPCR positive for *Spn* colonization n = (82, 10.1%)	p-value
**Characteristic**				
Age mean, S.D.	62±15	62±15	64±15	0.39
Gender female, n (%)	394 (48.6)	357 (49.0)	37 (45.1)	0.50
Health Care Worker, n (%)	62 (7.7)	57 (7.8)	5 (6.1)	0.58
**Lives in the following condition, n (%)**				
Geriatric Home	16 (2.0)	16 (2.2)	0 (0.0)	0.18
With Small Children	106 (13.1)	97 (13.3)	9 (11.0)	0.55
Overcrowded	36 (4.4)	31 (4.3)	5 (6.1)	0.44
**Habits, n (%)**				
Alcoholic	1 (0.1)	1 (0.1)	0 (0.0)	0.74
Smoker	117 (14.4)	105 (14.4)	12 (14.6)	0.96
PAS	1 (0.1)	1 (0.1)	0 (0.0)	0.74
**Hematic Compromise, n (%)**				
Anemia	10 (1.2)	9 (1.2)	1 (1.2)	1.00
**Immune System Compromise, n (%)**				
Immunologic Compromise^+^	139 (17.2)	132 (18.1)	7 (8.5)	0.03
**Neurologic Compromise, n (%)**				
Stroke	8 (1.0)	8 (1.1)	0 (0.0)	0.34
Dementia	4 (0.5)	4 (0.5)	0 (0.0)	0.50
Other Neurologic Disease	33 (4.1)	32 (4.4)	1 (1.2)	0.17
**Hepatic Compromise, n (%)**				
Chronic Hepatic Disease**	11 (1.4)	9 (1.2)	2 (2.4)	0.37
**Pulmonary Compromise, n (%)**				
Pulmonary Disease	69 (8.5)	59 (8.1)	10 (12.2)	0.21
**Renal Compromise, n (%)**				
Chronic Kidney Disease	141 (17.4)	125 (17.2)	16 (19.5)	0.60
Renal Replacement Therapy	137 (16.9)	122 (16.8)	15 (18.3)	0.73
**Cardiovascular Compromise, n (%)**				
Hypertension	423 (52.2)	381 (52.3)	42 (51.2)	0.85
Cardiac Disease*	252 (31.1)	219 (30.1)	33 (40.2)	0.06
**Other, n (%)**				
Catheter probe	26 (3.2)	26 (3.6)	0 (0.0)	0.26
Tracheostomy	1 (0.1)	1 (0.1)	0 (0.0)	0.06
**Outcomes, n (%)**				
Unfavorable outcome	203 (25.0)	179 (24.5)	24 (29.2)	0.13
Hospitalizations^•^^‣^	142 (17.5)	128 (17.5)	14 (17.0)	0.93
Deaths^•^	58 (7.1)	47 (6.4)	11 (13.4)	<0.01
Pneumonia^•^	30 (3.7)	28 (3.8)	2 (2.4)	0.45

**Abbreviations:** q-rtPCR (quantitative—real time polymerase chain reaction), S.D. (Standard deviation), P.A.S. (psychoactive substances), C.O.P.D. (Chronic obstructive pulmonary diseases), O.H.S.A.S. (obstructive hypopnea sleep apnea syndrome).

* Includes heart failure, coronary disease, myocardial infarction, and arrhythmia.

^+^ Includes recent transplants, cancer, rheumatoid arthritis, lupus, other autoimmune disease, H.I.V infection, AIDS, chemotherapy, biological therapy, and leukopenia by any cause.

** Includes chronic hepatic diseases such as viral or autoimmune hepatitis and cirrhosis.

^•^ Included within the composite variable “unfavorable outcome”.

^‣^ Due to an infectious cause.

Regarding vaccination, 31.6% (256/810) of patients had received the influenza vaccine during the prior year, while 10.7% (87/810) had been administered a version of the anti-*Spn* vaccine before study enrollment. Among those who received the pneumococcal vaccine, 50% (44/87) were administered the PPSV-23, 11.4% (10/87) received PCV-13, and 3.4% (3/87) were given the PSSV+PCV scheme. Notably, 33.3% (29/87) of individuals could not specify the type of pneumococcal vaccine they received but were aware of having received it. Only 1.1% (1/87) reported receiving PCV-10. Further details regarding vaccination within each subgroup based on local guidelines can be found in (Table 2).

**Table 2:** Vaccination rates by comorbidity included in local adult vaccination guideline indications.

**Total population n = 810**	**60 years or older n = 258**	**Pulmonary disease n = 69**	Cardiac disease* **n = 252**	**Immunologic compromise n = 258**	**Cirrhotic n = 2**	**Diabetic n = 125**	**Smoker n = 117**	**Alcoholic n = 1**	**Asplenia n = 0**	**RRT n = 137**
**Pneumococcal vaccine ratio n (%)**	87/258 (33)	9/69 (13)	43/252 (17)	36/258 (13.9)	0/2 (0)	18/125 (14.4)	12/117 (10.2)	0/1 (0)	0/0 (0)	24/137 (17.5)
**Total population n = 810**	**60 years or older n = 258**	**Pulmonary disease n = 69**	Cardiac disease* **n = 252**	**Immunologic compromise**^+^ **n = 139**	**CHD**^++^ **n = 11**	**Diabetic n = 125**	**RRT n = 137**	**Morbid obesity** ^Δ^ **n = 0**		
**Influenza vaccine ratio n (%)**	11/258 (38.7)	25/69 (36.2)	95/252 (37.6)	40/108 (37)	4/11 (36.3)	45/125 (36)	56/137 (40.8)	0/0 (0)		

**Abbreviations**: CHD (Chronic hepatic disease), RRT (Renal replacement therapy).

* Includes heart failure, coronary disease, myocardial infarction, and arrhythmia.

^+^ Includes recent transplants, cancer, rheumatoid arthritis, lupus, other autoimmune disease, H.I.V., AIDS, chemotherapy, biological therapy and leukopenia by any cause.

^++^ Includes chronic hepatic diseases such as viral or autoimmune hepatitis and cirrhosis.

^Δ^ Cutoff point of 40 kg/m^2^ body mass index was used to identify these patients.

**Fig 1:**
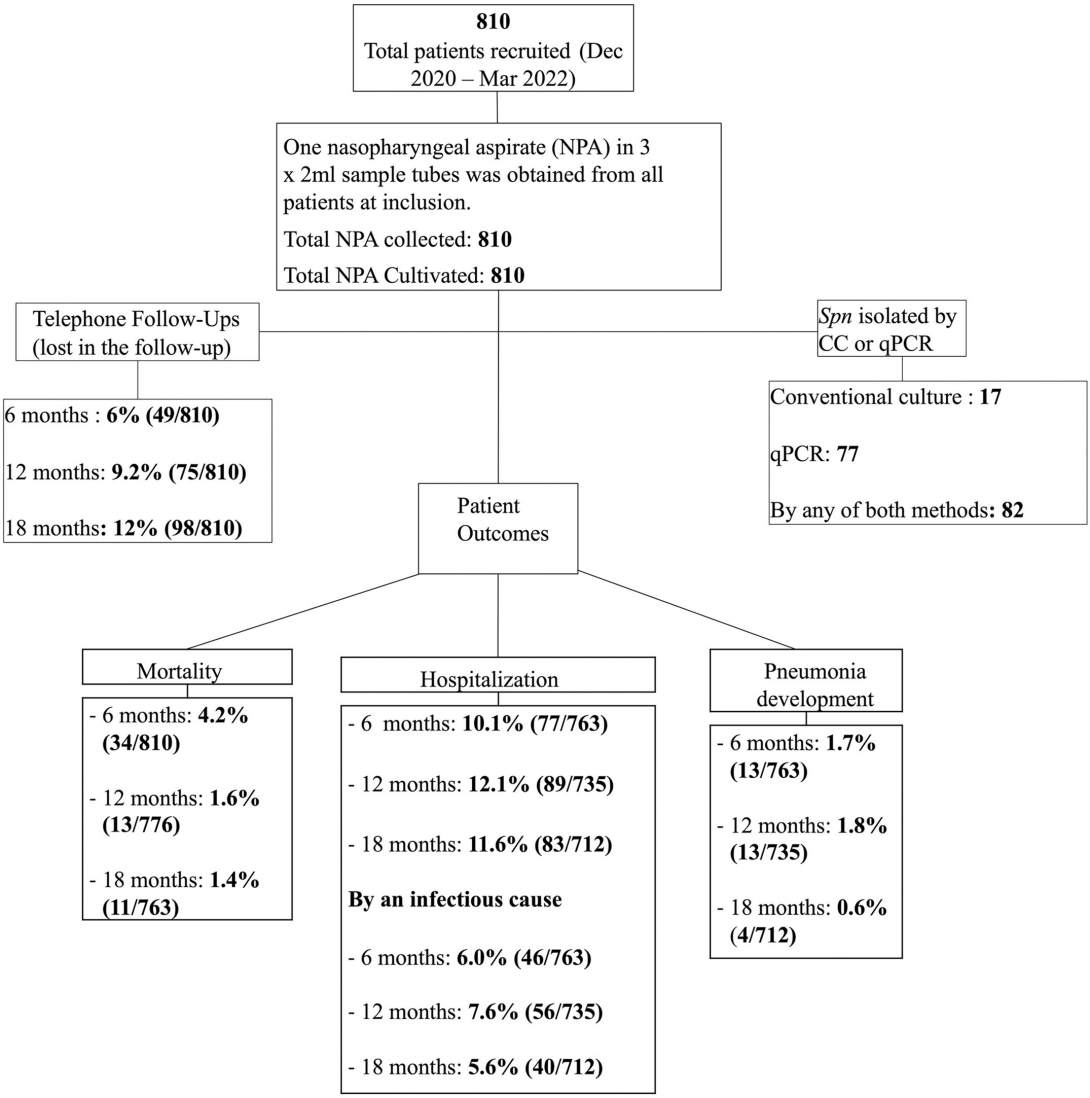
*Spn* isolated and patients’ unfavorable outcomes during the 18-month follow-up period. Flowchart describing the number of samples collected (NPA: Nasopharyngeal Aspirate), the analysis method (CC: Conventional Culture, q-rtPCR: Real-time Polymerase Chain Reaction), the follow-up phases, the proportion of subjects lost during the study period, and the percentages of unfavourable outcomes, including hospitalization, pneumonia cases, and mortality in each phase.

Over the 18-month follow-up period, 3.7% (30/810) of the cohort developed pneumonia, comprising 13 cases at 6 months, 13 at 12 months, and 4 cases at 18 months. In total, 20.2% (164/810) of participants were hospitalized for any cause during the follow-up, resulting in 248 admissions: 76 at 6 months, 89 at 12 months, and 83 at 18 months. Among these hospitalizations, 46 were attributed to infectious causes at 6 months, 56 at 12 months, and 40 at 18 months, contributing to 142 hospitalizations included in the composite outcome variable of unfavorable outcome. The mortality rate was 4.1% (34/810) at 6 months, 1.7% (13/776) during the following 6 months, and reached 1.4% (11/763) during the last 6 months for a total of 7.2% (58/810) during the entire 18-month follow-up period. Additional insights into the observed outcomes at each time point are visually presented in (Fig 1), providing a comprehensive overview of the cohort’s trajectory over the specified period.

### Outcomes

**Fig 2:**
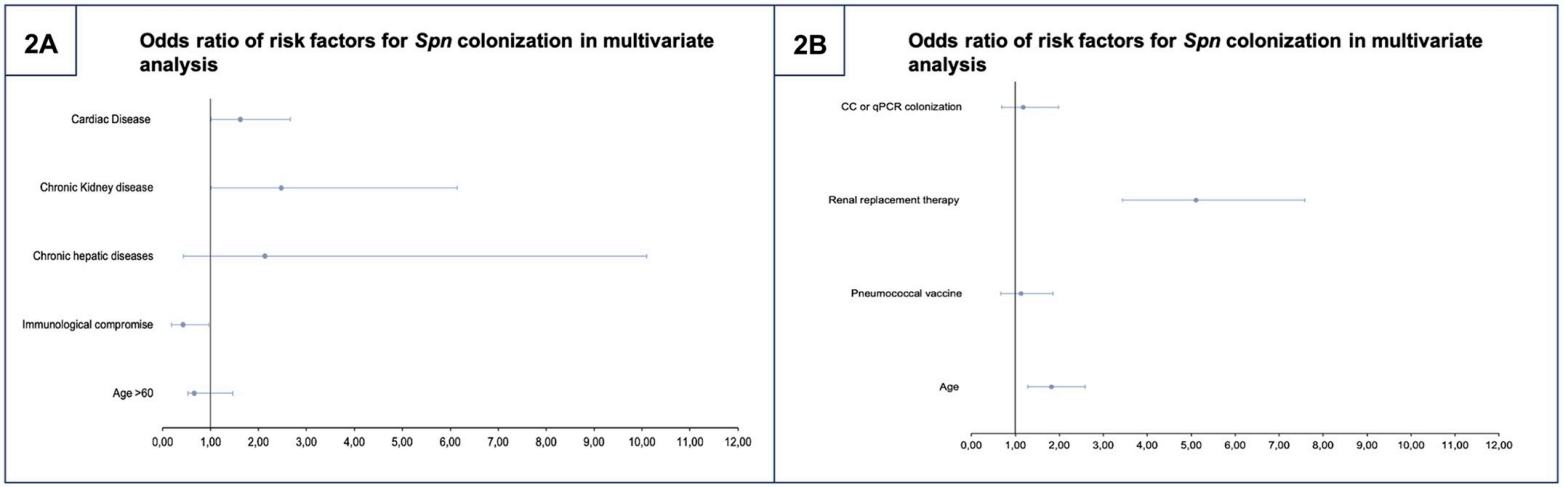
Multivariate analysis of risk factors for *Spn* colonization and unfavorable outcomes. Forrest plots of multivariate analysis for colonization risk factors and its impact on unfavorable outcomes (2A) Risk factors for *Spn* Colonization. (2B) Risk factors for unfavorable outcomes.

The *Spn* NPC period prevalence was 10.1% (82/810) during the patient observation period. Over the 18-month follow-up, 25% (203/810) of patients developed any of the three considered outcomes within the unfavorable outcome variable. A multivariate logistic regression was conducted to explore the association between comorbidities and *Spn* colonization. In this analysis, chronic kidney disease was identified as a risk factor for colonization, showing an odds ratio (OR, [95% CI]) of 2.48 (1.01–6.15), *p* = 0.04. Similarly, cardiac diseases presented a statistically significant risk factor, albeit crossing the confidence interval, with an OR [95% CI] of 1.62 (0.99–2.66), *p* = 0.05. Although significant in the univariate analysis, chronic hepatic disease did not demonstrate an association in this multivariate analysis. Details on specific outcomes for the univariate and multivariate analysis can be found in (Fig 2A). Hosmer-Lemeshow test for binary logistic regression models demonstrated the goodness-of-fit test (p = 0.703).

Similarly, a multivariate binary logistic regression analysis was conducted to examine whether colonization by *Spn* predisposed patients to develop an unfavorable outcome, as previously defined. This analysis, adjusted for age, vaccination status, and the need for renal replacement therapy, revealed that colonization by either traditional culture or q-rtPCR showed no statistically significant association with unfavorable outcomes (OR [95% CI]) of 1.17 (0.69–1.98), *p* = 0.54. Further information regarding both univariate and multivariate analyses can be found in the online supplement (S3 and S4 Tables). Moreover, a sub-analysis specifically for LTRI development showed that colonization was not a risk factor for LTRI development, taking into account the bias that the low number of events represent (OR [95% CI]) of 0.64 (0.14–2.78), *p* = 0.55 (S5 Table). Age was demonstrated to be independently associated with unfavorable outcomes in the multivariate analysis (1.81 [1.28–2.58], *p* < 0.01). As expected, the need for renal replacement therapy emerged as a significant contributor to unfavorable outcomes in the multivariate analysis (5.10 [3.43–7.58], *p* < 0.01). Specific outcomes from the univariate analysis are detailed in (Fig 2B), and the Hosmer-Lemeshow test for binary logistic regression models demonstrated the goodness-of-fit test (*p* = 0.548).

## Discussion

The reported *Spn* colonization of 10.1% (82/810) in the present research aligns with findings from prior studies in adults, which have reported a prevalence range of 1–10% [20, 21]; the most recent metanalysis found an overall prevalence of 6% and in the subgroup analysis a prevalence of 2% for adults [26]. Previous research on adult colonization varies in methodology, age range, and sample size, often lacking comorbidity assessments. Abdullahi et al. presented a similar study reported in Kenya in age range. Still, they did not consider comorbidities, reporting a rate of 6.4% (n = 450) in adults >18 years [27], and Palmu et al. reported a 5,23% prevalence in healthy adults >65 years (n = 592) [28]. Conversely, comorbidity-focused studies, such as those published by Heinsbroek et al. and Dayie et al. in Malawi and Ghana, show higher rates (21.3%, 10.0%) in adults with HIV and sickle cell anemia respectively [29, 30]. Studies made by Milucky et al. and Roca-Oporto et al. conducted in high-income countries like the US and Spain reported lower rates (1.8% to 5,6%) in individuals with solid organ transplants and chronic diseases [31, 32]. Notably, patients with influenza-related respiratory symptoms exhibit rates as high as 31% [33]. However, even though Colombia is classified as an upper-middle-income country, this study identified a *Spn* colonization rate of 5.0% (7/139) in patients with immunocompromise, which is consistent with the rates described by Milucky et al. and Roca-Oporto et al. [31, 32]. Therefore, the presented findings are novel because they assessed the nasopharyngeal colonization by *Spn* in a broader cohort of patients with multiple chronic comorbid conditions, using traditional cultures and q-rtPCR based technics, making these results more generalizable.

This real-world study found a low anti-*Spn* vaccination rate among adults with comorbid conditions in Colombia, which agrees with previous studies conducted in Colombia by Severiche et al. and Serrano et al. [4, 34]. Also, the current study found a low colonization rate despite suboptimal vaccination rates per local guidelines. In contrast, prior literature had shown that before the introduction of the conjugate vaccine in state schedules, proportions of culture-based colonization in HIV adult patients were as high as 16% and 18% in Brazil and Uganda, as described by Nicoletti et al. and Blossom et al. [35, 36]. Becker-Dreps et al. found in older adults with general comorbidity a 1.9% colonization rate before the introduction of anti-*Spn* vaccines in the US [37]. Following the incorporation of conjugate vaccines (PCV 7, 10, 13, 15, and 20), Drayss were found in adults > 65 years from geriatric homes in Germany with low comorbidity levels and colonization rates of 0% [38]. Even though the role that plays anti-*Spn* vaccines in nasopharyngeal colonization has been controversial, and different factors might affect the colonization rates, this study´s results reinforce the need for more robust vaccination campaigns in adults with chronic comorbid conditions, as it has been described that solid vaccination campaigns could reduce IPD development in adults [39].

Van Hoek and Pekuz et al. studies have indicated a relationship between CKD and IPD [40, 41]. Also, some studies have highlighted better outcomes and survival rates in vaccinated patients with comorbid conditions [42, 43]. However, limited research explores CKD’s link to colonization. Cardiovascular disease (CVD) and heart failure are frequently associated with pneumococcal disease [44–46], but their association with colonization risk remains uncertain across studies [47]. Thus, identifying CKD and chronic cardiac diseases independently associated with nasopharyngeal *Spn*-colonization, highlights the importance of vaccination in these groups of patients.

Finally, the present study did not find an association between *Spn* colonization and the development of any of the three unfavorable outcomes, which contradicts the currently available data on children. This finding is novel as, to our knowledge, this study is the first study exploring this relationship in adults with comorbid conditions. One possible explanation for this study´s findings and how they differ from what has been previously reported in children is that adults have, as a result of prior exposure events to *Spn*, generated an adaptive immune response to *Spn* proteins, which would confer broad protection from disease caused by all serotypes of *Spn*. Alternatively, during childhood, these individuals were colonized by versions of *Spn* that carry capsule types most frequently associated with disease, and now, due to the production of antibodies to these capsule types, they are more likely to be colonized by strains not as capable of causing severe infection. Therefore, further studies are needed to understand the etiology of LRTI and unfordable outcomes in patients with comorbid conditions.

The present study has some limitations that should be mentioned. First, this cohort size is relatively small, potentially impacting the ability to draw statistically significant associations. In addition, only one NPA sampling was performed, which, given the dynamics of *Spn* colonization, does not allow us to adequately determine whether there is an association between *Spn* NPC and the development of unfavorable outcomes. However, the study was powered to identify statistical differences, and more importantly, to the best of our knowledge, this is the first study assessing nasopharyngeal colonization in adults with comorbidities, rendering these findings applicable to a significant demographic subset. Second, this study found a low prevalence of pneumococcal colonization that could be attributed to the inherent difficulty in culturing *Spn*. Nevertheless, the study employed molecular tests to augment the likelihood of successful isolations, enhancing the precision of the analysis. Furthermore, this study overlapped with the COVID-19 pandemic, which may have impacted social dynamics, underestimating the actual behavior of the colonization phenomena [48].

In conclusion, this novel study reinforces existing evidence that comorbidities such as CKD and cardiac diseases increase susceptibility to pneumococcal colonization. These findings underscore the imperative need for vaccination in this population. Vaccination rates, particularly for pneumococcal vaccines, were suboptimal, highlighting the urge to implement adherence to vaccination guidelines. Finally, this research found that nasopharyngeal colonization by *Spn* was not associated with unfavorable outcomes in this population, including the development of LRTI. There is a need for further research to understand the complex interactions between colonization, comorbidities, and clinical outcomes.

## Supporting information

S1 Table Comorbidities included in the study and their definition. (PDF)

S2 Table Heterogeneity amongst colonized and non-colonized groups of patients. (PDF)

S3 Table Univariate and multivariate analysis for factors associated with colonization. (PDF)

S4 Table Univariate and multivariate analysis for colonization as a risk factor for unfavorable outcomes. (PDF)

S5 Table *Spn* NPC as a risk factor for LTRI, multivariate analysis. (PDF)

S1 File Materials, methods, and protocols used in the study. (PDF)
