# Supplementary material for: The relation of nasopharyngeal colonization by Streptococcus pneumoniae in comorbid adults with unfavorable outcomes in a low-middle income country
Source: PLoS One. 2025 Feb 12;20(2):e0318320. doi: 10.1371/journal.pone.0318320 (PMC11819510; doi:10.1371/journal.pone.0318320)
Supplement: S1 Table — (PDF) [file pone.0318320.s001.pdf]

Supplementary material 1. Comorbidities included in the study and their definition.

Comorbidity	Type of variable	Description
Alcoholism	Comorbidities – dichotomic	A primary, chronic disease with genetic, psychosocial, and environmental factors influencing its development and manifestations. The disease is often progressive and fatal. It is characterized by impaired control over drinking, preoccupation with the drug alcohol, use of alcohol despite adverse consequences, and distortions in thinking, most notably denial. Each of these symptoms may be continuous or periodic. (Morse and Flavin for the Joint Commission of the National Council on Alcoholism and Drug Dependence and the American Society of Addiction Medicine to Study the Definition and Criteria for the Diagnosis of Alcoholism: in JAMA 1992;268:1012-4)
Asplenia	Comorbidities – dichotomic	Congenital or acquired absence of the spleen (for example, after surgical removal).
Stroke	Comorbidities – dichotomic	A group of pathological conditions characterized by sudden, non-convulsive loss of neurological function due to BRAIN ISCHEMIA or INTRACRANIAL HEMORRHAGES. Stroke is classified by the type of tissue NECROSIS, such as the anatomic location, vasculature involved, etiology, age of the affected individual, and hemorrhagic vs. non-hemorrhagic nature. (From Adams et al., Principles of Neurology, 6th ed, pp777-810)
COPD	Comorbidities – dichotomic	A disease of chronic diffuse irreversible airflow obstruction. Subcategories of **COPD** include CHRONIC BRONCHITIS and PULMONARY EMPHYSEMA.
Anemia	Comorbidities – dichotomic	A reduction in the number of circulating ERYTHROCYTES or in the quantity of HEMOGLOBIN.
Transplant	Comorbidities – dichotomic	Organs, tissues, or cells taken from the body for grafting into another area of the same body or into another individual.

Bronchiectasis	Comorbidities – dichotomic	Persistent abnormal dilatation of the bronchi.
Cancer	Comorbidities – dichotomic	Cancer is not just one disease, but a large group of almost 100 diseases. Its two main characteristics are uncontrolled growth of the cells in the human body and the ability of these cells to migrate from the original site and spread to distant sites. If the spread is not controlled, cancer can result in death.
Cirrhosis	Comorbidities – dichotomic	Liver disease in which the normal microcirculation, the gross vascular anatomy, and the hepatic architecture have been variably destroyed and altered with fibrous septa surrounding regenerated or regenerating parenchymal nodules
Dementia	Comorbidities – dichotomic	An acquired organic mental disorder with loss of intellectual abilities of sufficient severity to interfere with social or occupational functioning. The dysfunction is multifaceted and involves memory, behavior, personality, judgment, attention, spatial relations, language, abstract thought, and other executive functions. The intellectual decline is usually progressive, and initially spares the level of consciousness.
Diabetes	Comorbidities – dichotomic	A heterogeneous group of disorders characterized by HYPERGLYCEMIA and GLUCOSE INTOLERANCE.
Chronic hepatic	Comorbidities – dichotomic	Sudden liver failure in the presence of underlying compensated chronic LIVER DISEASE (e.g., LIVER CIRRHOSIS; HEPATITIS; and liver injury and failure) due to a precipitating acute hepatic insult
mental disease	Comorbidities – dichotomic	Psychiatric illness or diseases manifested by breakdowns in the adaptational process expressed primarily as abnormalities of thought, feeling, and behavior producing either distress or impairment of function
chronic kidney	Comorbidities – dichotomic	The end-stage of CHRONIC RENAL INSUFFICIENCY. It is characterized by the severe irreversible kidney damage (as measured by the level of PROTEINURIA) and the reduction in GLOMERULAR FILTRATION RATE to less than 15 ml per min (Kidney Foundation: Kidney Disease Outcome Quality Initiative, 2002). These

		patients generally require HEMODIALYSIS or KIDNEY
neurologic disease	Comorbidities – dichotomic	Diseases of the central and peripheral nervous system. This includes disorders of the brain, spinal cord, cranial nerves, peripheral nerves, nerve roots, autonomic nervous system, neuromuscular junction, and muscle
arrhythmia	Comorbidities – dichotomic	Any disturbances of the normal rhythmic beating of the heart or MYOCARDIAL CONTRACTION. Cardiac arrhythmias can be classified by the abnormalities in HEART RATE, disorders of electrical impulse generation, or impulse conduction
coronary disease	Comorbidities – dichotomic	An imbalance between myocardial functional requirements and the capacity of the CORONARY VESSELS to supply sufficient blood flow. It is a form of MYOCARDIAL ISCHEMIA (insufficient blood supply to the heart muscle) caused by a decreased capacity of the coronary vessels.
heart failure	Comorbidities – dichotomic	A heterogeneous condition in which the heart is unable to pump out sufficient blood to meet the metabolic need of the body. **Heart failure** can be caused by structural defects, functional abnormalities (VENTRICULAR DYSFUNCTION), or a sudden overload beyond its capacity. Chronic **heart failure** is more common than acute **heart failure** which results from sudden insult to cardiac function, such as MYOCARDIAL INFARCTION.
Hypertension	Comorbidities – dichotomic	Persistently high systemic arterial BLOOD PRESSURE. Based on multiple readings (BLOOD PRESSURE DETERMINATION), **hypertension** is currently defined as when SYSTOLIC PRESSURE is consistently greater than 140 mm Hg or when DIASTOLIC PRESSURE is consistently 90 mm Hg or more.
Rheumatoid arthritis	Comorbidities – dichotomic	A chronic systemic disease, primarily of the joints, marked by inflammatory changes in the synovial membranes and articular structures, widespread fibrinoid degeneration of the collagen fibers in mesenchymal tissues, and by atrophy and rarefaction of

		bony structures. Etiology is unknown, but autoimmune mechanisms have been implicated.
Lupus	Comorbidities – dichotomic	A chronic, relapsing, inflammatory, and often febrile multisystemic disorder of connective tissue, characterized principally by involvement of the skin, joints, kidneys, and serosal membranes. It is of unknown etiology, but is thought to represent a failure of the regulatory mechanisms of the autoimmune system. The disease is marked by a wide range of system dysfunctions, an elevated erythrocyte sedimentation rate, and the formation of L.E. cells in the blood or bone marrow
autoimmune disease	Comorbidities – dichotomic	Disorders that are characterized by the production of antibodies that react with host tissues or immune effector cells that are autoreactive to endogenous peptides.
Renal Replacement Therapy (RRT)	Comorbidities – dichotomic	Procedures which temporarily or permanently remedy insufficient cleansing of blood metabolites by the kidneys
obesity	Comorbidities – dichotomic	A status with BODY WEIGHT that is grossly above the recommended standards, usually due to accumulation of excess FATS in the body. The standards may vary with age, sex, genetic or cultural background. In the BODY MASS INDEX, a BMI greater than 30.0 kg/m2 is considered obese, and a BMI greater than 40.0 kg/m2 is considered morbidly obese (MORBID OBESITY)
supplemental oxygen	Comorbidities – dichotomic	Supplemental oxygen is commonly used in modern medicine and is fundamental to the treatment of hypoxemia. Patients in an acute care setting through a nasal cannula, simple face mask, face tent, nonrebreather face mask, Venturi mask, and high-f low nasal cannula.
Sleep Apnea Syndromes	Comorbidities – dichotomic	Disorders characterized by multiple cessations of respirations during sleep that induce partial arousals and interfere with the maintenance of sleep. Sleep apnea syndromes are divided into central (see SLEEP APNEA, CENTRAL), obstructive (see SLEEP APNEA, OBSTRUCTIVE), and mixed central-obstructive types

HIV	Comorbidities – dichotomic	Human immunodeficiency virus. A non-taxonomic and historical term referring to any of two species, specifically HIV-1 and/or HIV-2. Prior to 1986, this was called human T-lymphotropic virus type III/lymphadenopathy-associated virus (HTLV-III/LAV). From 1986-1990, it was an official species called HIV Since 1991, HIV was no longer considered an official species name; the two species were designated HIV-1 and HIV-2.
Aids	Comorbidities – dichotomic	An acquired defect of cellular immunity associated with infection by the human immunodeficiency virus (HIV), a CD4-positive T-lymphocyte count under 200 cells/microliter or less than 14% of total lymphocytes, and increased susceptibility to opportunistic infections and malignant neoplasms. Clinical manifestations also include emaciation (wasting) and dementia. These elements reflect criteria for AIDS as defined by the CDC in 1993
drug addiction	Comorbidities – dichotomic	Disorders related to substance use or abuse.
Smoking	Comorbidities – dichotomic	Willful or deliberate act of inhaling and exhaling SMOKE from burning substances or agents held by hand
leukopenia	Comorbidities – dichotomic	A decrease in the number of LEUKOCYTES in a blood sample below the normal range (LEUKOCYTE COUNT less than 4000).
Chemotherapy	Comorbidities – dichotomic	Drug therapy given to augment or stimulate some other form of treatment such as surgery or radiation therapy. Adjuvant chemotherapy is commonly used in the therapy of cancer and can be administered before or after the primary treatment.

4
