# Supplementary material for: The relation of nasopharyngeal colonization by Streptococcus pneumoniae in comorbid adults with unfavorable outcomes in a low-middle income country
Source: PLoS One. 2025 Feb 12;20(2):e0318320. doi: 10.1371/journal.pone.0318320 (PMC11819510; doi:10.1371/journal.pone.0318320)
Supplement: S2 Table — (PDF) [file pone.0318320.s002.pdf]

Supplementary material 3. Heterogeneity amongst colonized and non-colonized groups of patients.

	Total patients sampled n=(810)	Conventional Culture or q-rtPCR negative for *Spn* colonization n=(728, 89.9%)	Conventional Culture or q-rtPCR positive for *Spn* colonization n=(82, 10.1 %)	p-value
**Characteristic**				
Age mean, S.D.	62±15	62±15	64±15	0.39
Gender female n,(%)	394 (48.6%)	357 (49.0%)	37 (45.1%)	0.50
Health Care Worker n,(%)	62 (7.7%)	57 (7.8%)	5 (6.1%)	0.58
**Lives in the following condition**				
Geriatric Home n, (%)	16 (2.0%)	16 (2.2%)	0 (0.0%)	0.18
With Small Children n,(%)	106 (13.1%)	97 (13.3%)	9 (11.0%)	0.55
Overcrowded n, (%)	36 (4.4%)	31 (4.3%)	5 (6.1%)	0.44
**Habits**				
Alcoholic n,(%)	1 (0.1%)	1 (0.1%)	0 (0.0%)	0.74
Smoker n,(%)	117 (14.4%)	105 (14.4%)	12 (14.6%)	0.96
PAS n,(%)	1 (0.1%)	1 (0.1%)	0 (0.0%)	0.74
**Hematic Compromise**				
Anemia n,(%)	10 (1.2%)	9 (1.2%)	1 (1.2%)	1.00
Leukopenia n,(%)	17 (2.1%)	17 (2.3%)	0 (0.0%)	0.16
**Immune System Compromise**				
Cancer n,(%)	37 (4.6%)	36 (4.9%)	1 (1.2%)	0.13
Chemotherapy n,(%)	13 (1.6%)	13 (1.8%)	0 (0.0%)	0.22
Transplant n,(%)	10 (1.2%)	10 (1.4%)	0 (0.0%)	0.29
Rheumatoid Arthritis n,(%)	33 (4.1%)	29 (4.0%)	4 (4.9%)	0.70
Lupus n,(%)	11 (1.4%)	11 (1.5%)	0 (0.0%)	0.26
Autoimmune Disease n,(%)	45 (5.6%)	41 (5.6%)	4 (4.9%)	0.78
Biological Therapy n,(%)	36 (4.4%)	35 (4.8%)	1 (1.2%)	0.14
Immunologic Compromise n,(%)	139 (17.2%)	132 (18.1%)	7 (8.5%)	0.03
**Neurologic Compromise**				
Stroke n,(%)	8. (1.0%)	8 (1.1%)	0 (0.0%)	0.34
Dementia n,(%)	4. (0.5%)	4 (0.5%)	0 (0.0%)	0.50

Other Neurologic Disease n,(%)	33. (4.1%)	32 (4.4%)	1 (1.2%)	0.17
**Hepatic Compromise**				
Chronic Hepatic Disease n,(%)	11. (1.4%)	9 (1.2%)	2 (2.4%)	0.37
Cirrhosis n,(%)	2 (0.2%)	1 (0.1%)	1 (1.2%)	0.06
All Hepatic Diseases n,(%)	11 (1.4%)	9 (1.2%)	2 (2.4%)	0.37
**Pulmonary Compromise**				
COPD n,(%)	67 (8.3%)	57 (7.8%)	10 (12.2%)	0.17
Bronchiectasis n,(%)	2 (0.2%)	2 (0.3%)	0 (0.0%)	0.64
OSAHS n,(%)	88 (10.9%)	81 (11.1%)	7 (8.5%)	0.47
Pulmonary Disease n,(%)	69 (8.5%)	59 (8.1%)	10 (12.2%)	0.21
**Renal Compromise**				
Chronic Kidney Disease n,(%)	141 (17.4%)	125 (17.2%)	16 (19.5%)	0.60
Renal Replacement Therapy n,(%)	137 (16.9%)	122 (16.8%)	15 (18.3%)	0.73
**Cardiovascular Compromise**				
Hypertension n,(%)	423 (52.2%)	381 (52.3%)	42 (51.2%)	0.85
Arrythmia n,(%)	79 (9.8%)	68 (9.3%)	11 (13.4%)	0.24
Myocardial Infarction n,(%)	125 (15.4%)	107 (14.7%)	18 (22.0%)	0.08
Coronary Disease n,(%)	176 (21.7%)	149 (20.5%)	27 (32.9%)	0.01
Heart Failure n,(%)	154 (19.0%)	129 (17.7%)	25 (30.5%)	0.01
Cardiac Disease n,(%)	252 (31.1%)	219 (30.1%)	33 (40.2%)	0.06

Abbreviations: qPCR (quantitative polymerase chain reaction), S.D. (Standard deviation), PAS (psychoactive substances), COPD (Chronic obstructive pulmonary diseases), O.H.S.A.S. (obstructive hypopnea sleep apnea syndrome).
