# Supplementary material for: The relation of nasopharyngeal colonization by Streptococcus pneumoniae in comorbid adults with unfavorable outcomes in a low-middle income country
Source: PLoS One. 2025 Feb 12;20(2):e0318320. doi: 10.1371/journal.pone.0318320 (PMC11819510; doi:10.1371/journal.pone.0318320)
Supplement: S3 Table — (PDF) [file pone.0318320.s003.pdf]

Supplementary material 4. Univariate and multivariate analysis for factors associated with colonization.

	Univariate Analysis		Multivariate Analysis	
**Variables**	**OR (95% CI)**	***p*-value**	**OR (95% CI)**	***p*-value**
Age >60	0.33 (0.53 - 1.46)	**0.33**	0.33 (0.53 - 1.46)	**0.63**
Gender	0.85 (0.29 - 1.96)	**0.56**		
Health care worker	0.76 (0.29 - 1.96)	**0.82**		
Lives in a geriatric home	0.97 (0.96 - 0.98)	**0.39**		
Lives overcrowded	1.4 (0.55 - 3.86)	**0.39**		
Pneumococcal vaccine*	0.88 (0.41 - 1.91)	**1.00**		
Influenza vaccine*	1.00 (0.615 - 1.67)	**1.00**		
Anemia	0.98 (0.12 - 7.88)	**1.00**		
immunologic compromise	0.42 (0.19 - 0.93)	0.03	0.43 (0.19 - 0.97)	**0.04**
Other neurologic diseases	0.026 (0.03 - 1.99)	0.24		
Chronic hepatic disease	1.99 (0.42 - 9.40)	**0.30**	2.13 (0.44 - 10.1)	**0.34**
Pulmonary disease	1.57 (0.77 - 3.21)	0.21		
Chronic kidney disease	1.16 (0.65 - 2.08)	0.64	2.48 (1.01 -6.15)	**0.04**
Renal replacement therapy	1.11 (0.61 - 2.01)	0.75		
Hypertension	0.95 (0.60 - 1.51)	0.90		
Cardiac disease	1.56 (0.97 - 2.50)	0.07	1.62 (0.99 - 2.66)	**0.05**
