# Supplementary material for: The relation of nasopharyngeal colonization by Streptococcus pneumoniae in comorbid adults with unfavorable outcomes in a low-middle income country
Source: PLoS One. 2025 Feb 12;20(2):e0318320. doi: 10.1371/journal.pone.0318320 (PMC11819510; doi:10.1371/journal.pone.0318320)
Supplement: S4 Table — (PDF) [file pone.0318320.s004.pdf]

Supplementary material 5. Univariate and multivariate analysis for colonization as a risk factor for unfavorable outcomes.

	Univariate Analysis		Multivariate Analysis	
**Variables**	**OR (95% CI)**	***p*-value**	**OR (95% CI)**	***p*-value**
Age	1.81 (1.28 - 2.58)	**<0.01**	1.81 (1.28 - 2.58)	**<0.01**
Pneumococcal vaccine*	1.45 (0.91 - 2.32)	**0.13**	1.12 (0.67 - 1.85)	0.65
immunologic compromise	1.11 (0.74 - 1.65)	0.6		
Stroke	0.83 (0.16 - 4.18)	**1.00**		
Dementia	2.53 (0.35 - 18.10)	**0.32**		
Other neurologic diseases	0.94 (0.43 - 2.06)	1.00		
Chronic hepatic disease	1.44 (0.42 - 4.99)	**0.51**		
Pulmonary disease	1.19 (0.70 - 2.04)	0.48		
Chronic kidney disease	1.71 (1.25 - 2.34)	<0.01		
Renal replacement therapy	5.08 (3.45 - 7.48)	<0.01	5.10 (3.43 - 7.58)	<0.01
Hypertension	1.71 (1.25 - 2.34)	<0.01		
Cardiac disease	1.41 (1.02 - 1.94)	0.04		
CC or qPCR colonization	1.19 (0.72 - 1.95)	0.51	1.17 (0.69 - 1.98)	0.54
