# Supplementary material for: The relation of nasopharyngeal colonization by Streptococcus pneumoniae in comorbid adults with unfavorable outcomes in a low-middle income country
Source: PLoS One. 2025 Feb 12;20(2):e0318320. doi: 10.1371/journal.pone.0318320 (PMC11819510; doi:10.1371/journal.pone.0318320)
Supplement: S5 Table — (PDF) [file pone.0318320.s005.pdf]

Supplementary material 6. *Spn* NPC as a risk factor for LTRI, multivariate analysis.

**Multivariate Analysis**		
**Variables**	**OR (95% CI)**	***p*-value**
Age >60	1.02 (0.98 - 1.45)	0.25
Pneumococcal vaccine	1.56 (0.57 – 4.28)	0.38
Renal replacement therapy	2.27 (0.99 – 5.17)	0.05
Colonization at any time	0.64 (0.14 – 2.78)	0.55
