# Supplementary material for: The relation of nasopharyngeal colonization by Streptococcus pneumoniae in comorbid adults with unfavorable outcomes in a low-middle income country
Source: PLoS One. 2025 Feb 12;20(2):e0318320. doi: 10.1371/journal.pone.0318320 (PMC11819510; doi:10.1371/journal.pone.0318320)
Supplement: S1 File — (PDF) [file pone.0318320.s006.pdf]

Supplementary material 2. Materials, methods and protocols used in the study.

Laboratory procedures

An immediate inoculation of 100 μL of NPA on blood agars were incubated at 37°C +/- 2°C for 24 - 48 hours in 5% CO2. Samples were aliquot in triplicate 2 mL cryovials, stored, and transported under refrigeration at 2 - 8° C for a maximum of 8 hours until transfer to the TSID-CCM research laboratory, where they were stored at – 80° C. Additionally, 100 μL of NPA was inoculated in another blood agar in the laboratory and incubated in conditions like those described above. The alpha-hemolytic colonies were selected and grew in blood agar under the previously mentioned conditions. Following this, they underwent the optochin sensitivity test and were cryopreserved at -80°C in thioglycolate broth containing 15% glycerol. Strains sensitive to optochin were further identified by performing Matrix Assisted Laser Desorption/Ionization Time-of-Flight Mass Spectrometry (MALDI-TOF) (B.D. Bruker, MALDI Biotyper).

In this way, we define "suspected case" as colonization with *Spn* by culture method when the presence > = 1 CFU/µL of alpha-hemolytic microorganisms and optochin sensitivity > 14 mm (15). Then, scores in MALDI-TOF > 1.8 were performed to determine a "confirmed case" (16). In addition, we decided to expand compare dcompare ncompare acompare pcompare xcompare ethods for identifying pneumococcal colonization using direct NPA. Therefore, all reference samples were tested with qPCR to detect amplification of two genes (ply and lytA) with sensitive, specific, and highly conserved primers for *S. pneumoniae (17-19)*. Information regarding primer selection, run cycle, and standard curve identification for ply and lytA gene identification can be found in the online supplement.

Primer selection:

We followed WHO guidelines, choosing primers for highly conserved genes like ply and lytA by amplification of a region-specific in genes through Quantitative PCR in Real Time (qPCR) using SYBR. Green - Universal Luna® Master mix (New England Biolabs) in machine CFX 96 Real-Time System (Biorad) (17).

Run cycle:

Each gene was independently analyzed. The run cycle was conducted as previously outlined with modifications: initial denaturation at 95°C for 10 minutes, followed by an amplification phase comprising 40 cycles at 95°C for 15 seconds, annealing at the melting temperature (Tm) of 64°C for 30 seconds. Subsequently, a melting cycle (ranging from 50 to 95°C with a 0.5°C increment every 2 seconds) was performed to assess the presence of polymorphisms or nonspecific aggregations in the amplifications. This assessment was based on the fluorescence dissociation characteristics due to temperature increment, utilizing a reference curve.

The data of the amplification was analyzed by Maestro CFX96 -Biorad software. The assay was performed in 15 µL. We used a 10 ng/ul DNA approximate concentration and a final concentration 0.5 – 0.1 nM primers concentration for the ply and lytA genes, respectively; in each running, a negative and positive control was included to validate the results.

Standard curve

A colony of an *S. pneumoniae* strain was suspended in 4 mL of saline solution (0.9% SSF) at a McFarland optical density of 0.5 (equivalent to a range of 0.08 to 0.1 absorbance at 550 nm), representing an initial concentration of 10^8 CFU/mL. Subsequently, serial dilutions were performed until reaching 10^1 CFU/mL. DNA extraction was carried out at each dilution to generate a standard curve. Finally, the bacterial viability was verified with culture in blood agar

of 100 µL the sample from the dilutions, and we determined the amount of DNA extracted by using a NanoDrop™ one - ThermoFishersupplPCR. The values obtained were traced in a standard curve.

Criteria for Colonization Identification

The definition of pneumococcal colonization using a combined approach of culture and real-time PCR necessitates adherence to specific criteria. These criteria encompass optochin sensitivity (halo > 14 mm), positive amplification of both genes (ply + lytA) with a sigmoidal amplification curve, a cycle threshold (Ct) < 35, and a delta fluorescence (dFUR) and delta temperature (dT) relationship in the melt curve akin to positive pneumococcal control samples (20). Amplifications with Ct > 35, a non-sigmoidal amplification curve, and a dissimilar melt curve profile compared to the positive control were considered nonspecific and negative for *Spn*.

Curve Standard melt and primer amplification curves for the gens ply and lytA PCR primers.

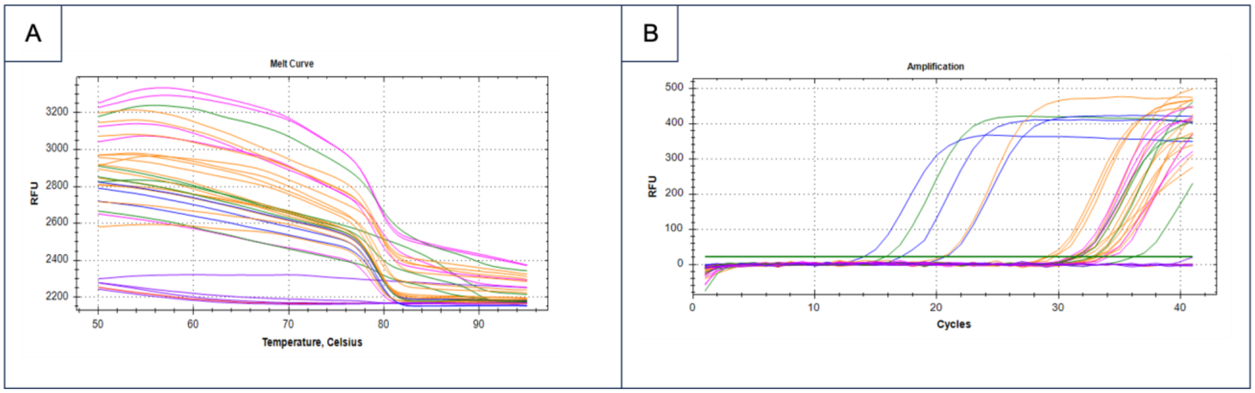

Curve standard_ gen ply

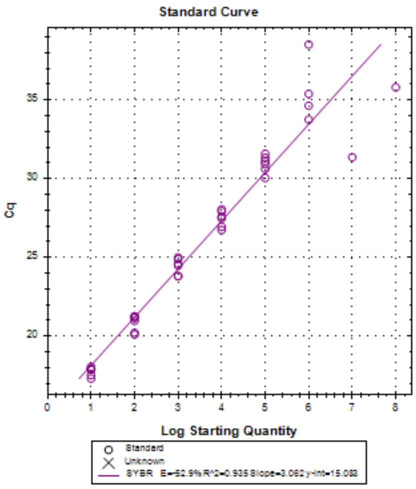

Curve amplification_ gen ply

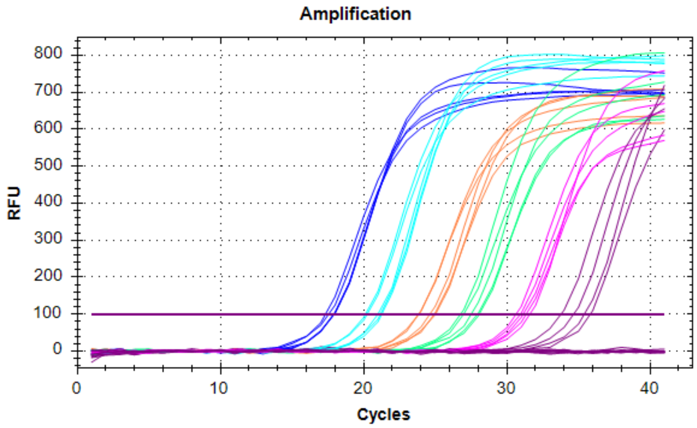

Curve standard _ gen lytA

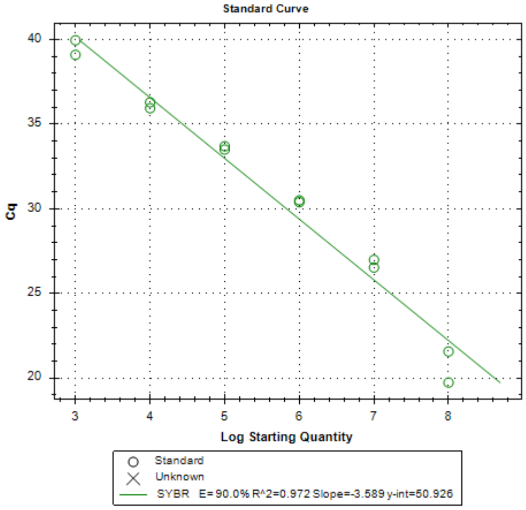

Curve amplification_ gen lytA

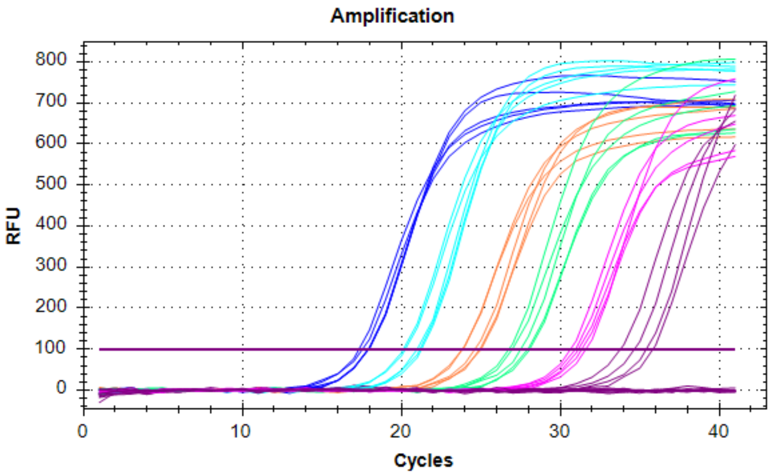
Amplification
RFU
800
700
600
500
400
300
200
100
0
0
10
20
30
40
Cycles
